# A porous proton-relaying metal-organic framework material that accelerates electrochemical hydrogen evolution

**DOI:** 10.1038/ncomms9304

**Published:** 2015-09-14

**Authors:** Idan Hod, Pravas Deria, Wojciech Bury, Joseph E. Mondloch, Chung-Wei Kung, Monica So, Matthew D. Sampson, Aaron W. Peters, Cliff P. Kubiak, Omar K. Farha, Joseph T. Hupp

**Affiliations:** 1 Department of Chemistry, Northwestern University, 2145 Sheridan Road, Evanston, Illinois 60208, USA; 2 Argonne-Northwestern Solar Energy Research (ANSER) Center, Northwestern University, Evanston, Illinois 60208, USA; 3 Department of Chemistry, Warsaw University of Technology, Noakowskiego 3, 00-664 Warsaw, Poland; 4 Department of Chemical Engineering, National Taiwan University, Number 1, Sector 4, Roosevelt Road, Taipei 10617, Taiwan; 5 Department of Chemistry and Biochemistry, University of California San Diego, 9500 Gilman Drive, La Jolla, California 92093-0358, USA; 6 Faculty of Science, Department of Chemistry, King Abdulaziz University, Jeddah 21589, Saudi Arabia; 7 Argonne National Laboratory, 9700 South Cass Avenue, Argonne, Illinois 60439, USA

## Abstract

The availability of efficient hydrogen evolution reaction (HER) catalysts is of high importance for solar fuel technologies aimed at reducing future carbon emissions. Even though Pt electrodes are excellent HER electrocatalysts, commercialization of large-scale hydrogen production technology requires finding an equally efficient, low-cost, earth-abundant alternative. Here, high porosity, metal-organic framework (MOF) films have been used as scaffolds for the deposition of a Ni-S electrocatalyst. Compared with an MOF-free Ni-S, the resulting hybrid materials exhibit significantly enhanced performance for HER from aqueous acid, decreasing the kinetic overpotential by more than 200 mV at a benchmark current density of 10 mA cm^−2^. Although the initial aim was to improve electrocatalytic activity by greatly boosting the active area of the Ni-S catalyst, the performance enhancements instead were found to arise primarily from the ability of the proton-conductive MOF to favourably modify the immediate chemical environment of the sulfide-based catalyst.

## Abstract (web summary)

Hydrogen evolution technologies for a future carbon-free energy economy require efficient catalysts which can be implemented on a large scale. Here, the authors prepare a composite electrode from readily available elements, whereby a metal-organic framework boosts catalytic performance by enabling rapid proton transport.

## Graphical abstract

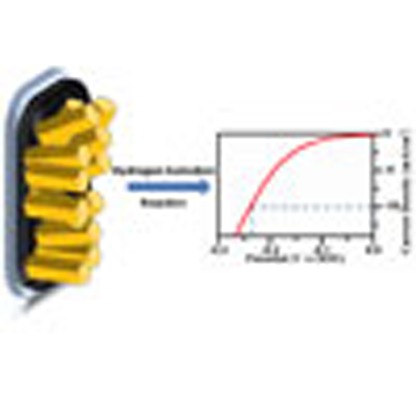

Substantial effort is being invested in the design and development of efficient electrocatalytic hydrogen evolution technologies, as these hold promise for a future carbon-free energy economy^1^,^2^,^3^,^4^. In acid solutions, the hydrogen evolution reaction (HER) entails the electrochemical reduction of protons: 2H^+^+2e^−^→H_2_. Catalysts are needed to decrease the otherwise enormous kinetic overpotential required to drive the reaction at suitably high current densities, *J*. For solar energy applications, where the corresponding oxidation reaction is the four-electron conversion of water to O_2_, a commonly cited target *J* value is 10 mA cm^−2^ (ref. 5). Provided that catalyst poisons are absent, selected noble metal electrodes (especially platinum electrodes) can support hydrogen evolution at high current densities under low kinetic overpotentials^6^. However, realization of large-scale hydrogen production requires the development of alternative low-cost electrocatalysts containing only highly abundant elements. A variety of candidate catalyst materials, including metal alloys^7^, carbides^8^, phosphides^9^,^10^, borides^8^ and nitrides^11^, have shown promising HER activity and stability in acidic solutions. In addition, several metal sulfides and selenides (MS/MS_2_/MSe/MSe_2_ where M=Co, W, Mo, Fe and Ni)^12^,^13^,^14^,^15^,^16^ have likewise recently been observed to display promising catalytic behaviour for hydrogen evolution. Nevertheless, substantial improvements in catalytic performance are needed to match the kinetic superiority of platinum electrodes.

Complementary to changing the composition of the electrocatalyst, a useful strategy for obtaining a significant leap in performance is to employ a porous supporting scaffold and create a composite electrode^17^,^18^. (A third approach, illustrated by various enzymes and their artificial mimics, is to modify the environment of the catalytically active site so as to provide proximal H-bond-donating or -accepting sites and/or proton-relay and -delivery channels)^19^,^20^,^21^. Porous scaffolds can boost an electrocatalyst's performance, that is, decrease overpotentials, by: (a) boosting the areal density of catalytically active surface sites, (b) improving the electronic conductivity of the electrode and/or (c) facilitating proton delivery. In addition, it has been shown that for some materials, certain crystal faces are much more catalytically active than others; the most striking examples perhaps are edge versus plane sites of layered, two-dimensional molybdenum or tungsten dichalcogenides^12^,^16^,^22^. By decreasing the lateral dimensions of crystallites constituting these catalysts, both a greater fraction and a greater absolute number of high-activity sites can be exposed^14^. The limit of this approach (not explored here) would be the synthesis of materials in small-cluster form, where the majority of exterior atoms, rather than only a tiny fraction, possesses the appropriate chemical coordination and geometric arrangement for high catalytic activity.

We reasoned that porous, electrode-surface-immobilized, metal-organic framework (MOF) crystallites might be ideal templates for synthesis of high-areal-density versions of inorganic electrocatalysts. In particular, we hypothesized that electrodeposition of nickel sulfide within the channels of the supported MOF might yield the desired composite or hybrid, nanostructured catalysts. Although underexplored as an HER electrocatalyst, especially in acidic environments, the electrodeposition of nickel sulfide is reasonably well understood^23^,^24^. Furthermore, a variety of sulfides of other first-row transition metals have been shown to be electrocatalytically competent, at modest kinetic overpotentials, for hydrogen evolution from water at low pHs^12^,^13^,^14^,^15^,^16^. As a candidate template or scaffold, we chose NU-1000 (ref. 25), a mesoporous MOF that can be grown solvothermally on conductive glass as a film comprising rod-shaped crystallites of a few microns length and a fraction of a micron width^26^. NU-1000 is one member of a growing family of porous MOFs featuring hexa-zirconium oxo/hydroxo/aquo clusters as nodes^27^,^28^,^29^,^30^,^31^ and displaying exceptional resistance to hydrolysis^32^,^33^,^34^, even at elevated temperature and/or in the presence of hydronium or hydroxide ions. Thus, this material was anticipated to be sufficiently chemically stable to function as a component of a hybrid HER electrocatalyst. It should be noted that various MOFs that anchor or encapsulate noble-metal nanoparticles or biomimetic molecular catalysts have recently been used in combination with molecular chromophores and sacrificial reductants (hole scavengers) to demonstrate photochemical hydrogen evolution^35^,^36^,^37^,^38^,^39^,^40^.

Here we report on the electrochemical assembly of a surface immobilized hybrid of Ni-S (we intentionally use Ni-S (rather than NiS) to denote a material comprising nickel and sulfur, but of initially unspecified metal:non-metal stoichiometry; we subsequently specify more precisely the composition of the form of Ni-S electrodeposited here) and NU-1000, and on the performance of the hybrid as an HER electrocatalyst in water at pH 1. The hybrid assembly yields a benchmark cathodic current of 10 mA cm^−2^ (together with H_2_ bubbles) at iR-corrected overpotentials (*η*-values) as low as 238 mV. Under the same conditions, and at the same benchmark current density, the observed overpotential with flat, MOF-free Ni-S films as electrocatalysts is 443 mV.

## Results

### Electrocatalyst synthesis

**Figure 1:**
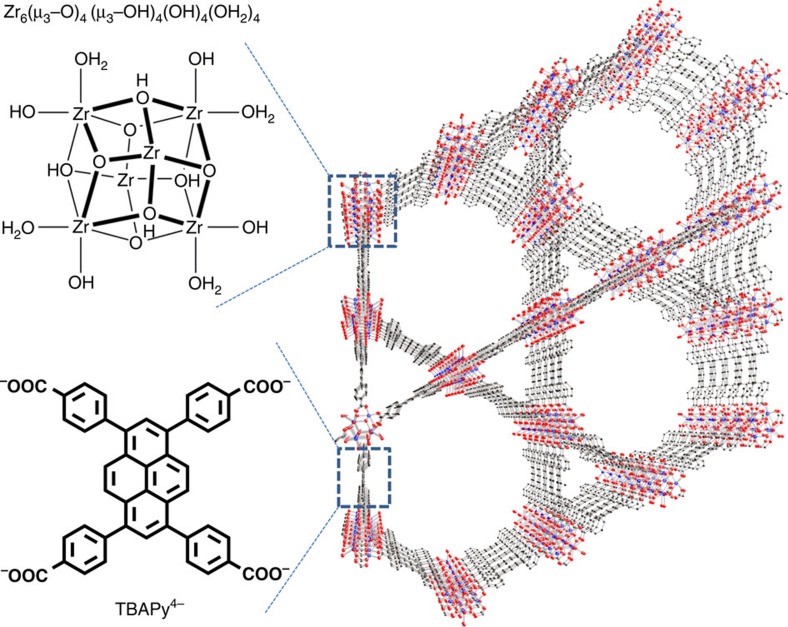
Schematic representation. Schematic representation of NU-1000's crystal structure.

**Figure 2:**
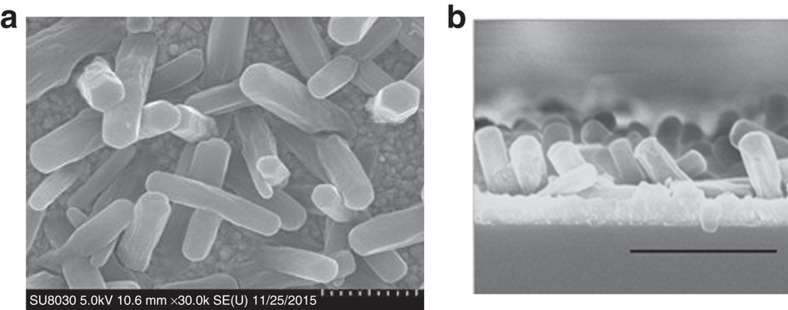
SEM images of NU-1000_Ni-S films. (**a**) SEM image of an NU-1000_Ni-S film showing the typical hexagonal rod-shaped crystals of NU-1000 on top of the FTO substrate (scale bar, 1 μm). (**b**) Cross-sectional SEM image of NU-1000_Ni-S film (scale bar, 2 μm).

Illustrations of the crystal structure of NU-1000, its Zr_6_(μ_3_–O)_4_(μ_3_–OH)_4_(OH)_4_(OH_2_)_4_ nodes^41^ and its organic linkers are presented in Fig. 1. The crystal structure shows the existence of both triangular and hexagonal one-dimensional channels; together with smaller apertures (*ca*. 8 Å diameter) along the channel walls, they are responsible for the high porosity of the material (the diameters of the one-dimensional channels are *ca*. 10 and 31 Å. In addition, around 20% of the hexagonal pores contain an extra node at the hexagon centre. The extra node is connected to the rest of the MOF via additional TBAPy^4−^ linkers. For simplicity, these are omitted from Fig. 1. N_2_ adsorption measurements (bulk samples) show that the MOF void volume is *ca*. 71% of the total volume). Figure 2a shows a scanning electron microscopy (SEM) image of a NU-1000_Ni-S electrode (2 min of Ni-S deposition, see also Supplementary Fig. 1 for experimental conditions), featuring the hexagonal rod-shaped crystals that are typical for NU-1000, whereas Fig. 2b shows a cross-sectional SEM view of a NU-1000_Ni-S electrode. X-ray Photoelectron Spectroscopy (XPS) measurements of the NU-1000_Ni-S film (Supplementary Fig. 2) confirm the presence of both nickel and sulfur. The observed S 2*p* binding energy is *ca*. 162 eV, implying sulfur incorporation as S^2−^. The presence of a peak at 168 eV is indicative of sulfur in a higher oxidation state, presumably oxy-sulfur species that might well arise from surface oxidation (air oxidation) of Ni-S^15^. The Ni 2*p* region shows a broad peak at a binding energy of centred around 860 eV, which can be attributed to Ni^2+^ as well as to other nickel oxidation states^42^.

**Figure 3:**
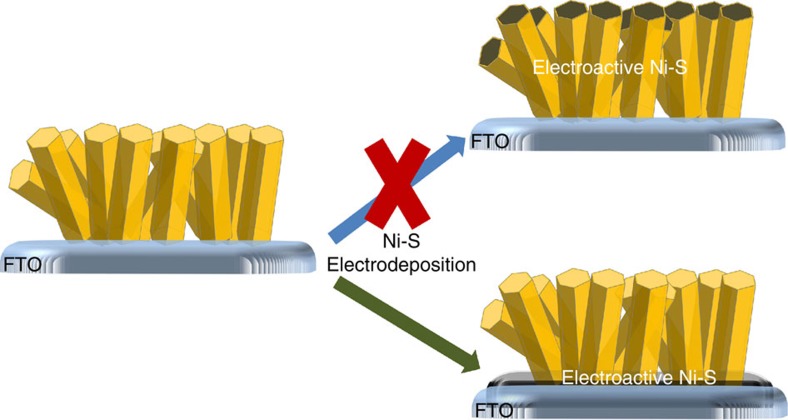
Illustration of Ni-S electrodeposition to create the NU-1000_Ni-S hybrid system. The initial aim was to use the MOF as a template for depositing high surface area Ni-S rods (top section). However, Ni-S was deposited as a flat layer (bottom section).

Elemental analysis of the hybrid films using both energy dispersive X-ray spectroscopy (EDS) and inductively coupled plasma-optical emission spectroscopy (ICP-OES), corroborate and quantify the conclusion that the hybrid films contain Ni and S. The EDS-obtained ratio of Ni:S was 1.7:1.0. ICP-OES yields a similar Ni:S ratio of 1.6:1.0, suggesting Ni_3_S_2_ as a primary deposition product (see Supplementary Table 1). EDS cross-sectional mapping of an MOF rod revealed, however, that only trace amount of nickel and sulfur are present in the extended portion of the MOF rods (Supplementary Fig. 3). Instead, these elements are confined and concentrated as a planar film, as illustrated in the bottom section of Fig. 3. As the MOFs themeslves are insulating in the region of potential where catalytic evolution of H_2_ occurs, the MOF-distributed traces of nickel and sulfur are electrochemically inaccessible and, therefore, unable to participate in electrocatalysis (HER electrocatalysis by metal sulfides generally requires proximal pairs of sulfur atoms (ions). The loading of sulfur in the extended region of the MOF, however, is <0.5 sulfur ions per hexa-zirconium node. Thus, even if the MOF could be rendered conductive, it is unlikely that these sulfur atoms would be catalytic).

Powder X-ray diffraction (XRD) measurements of putative FTO_NU-1000 films confirm that the observed crystallites consist of NU-1000 (Supplementary Fig. 4). Notably, the MOF's crystal structure remains intact. However, no peaks attributable to nickel sulfide are observed, suggesting that the material deposited is amorphous, in agreement with previous reports^23^.

More detailed information about the composition of the electrodeposited material was obtained via Raman spectroscopy. As can be seen in Supplementary Fig. 5, the Raman spectrum of a Ni-S MOF hybrid film contains the signature vibrational peaks of Ni_3_S_2_ (176, 190, 217, 300, 319 and 341 cm^−1^)^43^. In addition, it is important to note that small peaks are present, corresponding to NiS_2_ (ref. 44) (276, 447 and 465 cm^−1^) and NiS (379 cm^−1^)^45^. The Raman spectroscopy results combined with the EDS and ICP-OES elemental analysis establish that the main electrodeposition product is Ni_3_S_2_.

### HER catalysis

As suggested by the top portion of Fig. 3, our initial aim was simply to boost the metal-sulfide electrode surface area by using the MOF as a porous template for the electrodeposition of mesoscale nickel sulfide rods. In contrast to our expectations, however, the MOF did not function as a physical template and, consequently, did not increase the electrode surface area. Instead, Ni-S deposited as a flat film that in-filled the bottoms of the MOF rods, while coating both the bare conductive glass platform (fluorine-doped tin oxide, FTO) and the portions of FTO residing at the bottom of each MOF channel (see Fig. 3).

Notably, these channels are wide enough to readily allow permeation by the electrolyte solution in a hydrogen-evolution cell. As such, the MOF channels help to define the local chemical environment of the portion of the deposited Ni-S that is in contact with the electrolyte solution. As will be discussed further below, it is this local environment that alters the catalytic activity of the metal-sulfide electrode and yields the observed substantial decrease in HER overpotential.

**Figure 4:**
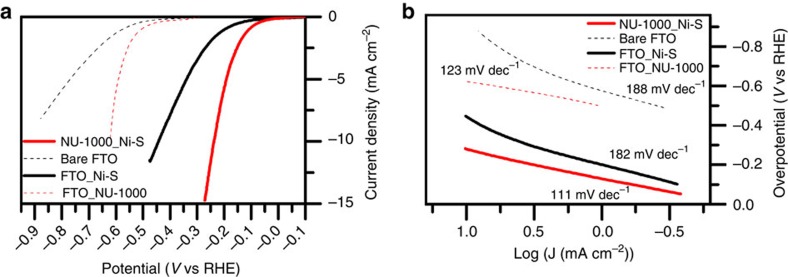
Comparison between the electrocatalytic HER performance. Four types of electrodes: bare FTO (dotted black), FTO_NU-1000 (dotted red), FTO_Ni-S (black) and NU-1000_Ni-S (red). (**a**) *J*–*V* curves. (**b**) Tafel plots. The listed values of 182 and 188 mV dec^−1^ for the HER Tafel slopes of the bare FTO and FTO_Ni-S samples refer to the low current density region only. The slopes clearly are larger at higher current densities.

To assess the electrocatalytic properties of the hybrid assembly towards hydrogen evolution, we studied four types of electrodes: bare FTO, a MOF scaffold grown on FTO substrate (FTO_NU-1000), Ni-S deposited on FTO substrate (FTO_Ni-S) and Ni-S deposited on FTO-supported NU-1000 (NU-1000_Ni-S). *J*–*V* curves and Tafel plots (*V* versus log *J*) of the different systems in aqueous 0.1 M HCl (pH 1) are shown in Fig. 4a. As expected, the bare FTO electrode exhibits poor electrocatalytic activity towards the HER, with a high reaction onset potential of around 600 mV versus Reversible Hydrogen Electrode (RHE) and an overpotential of *ca*. 1 V at *J*=10 mA cm^−2^. For Ni-S directly electrodeposited on FTO (only), the overpotential at 10 mA cm^−2^ is *ca*. 560 mV lower, clearly illustrating its ability to function as an electrocatalyst. Addition of the MOF scaffold, that is, formation of the hybrid assembly, further decreases the overpotential 10 mA cm^−2^ to 238 mV (this was the lowest iR-corrected overpotential observed based on several electrodes. The values occasionally ranged as a high as 280 mV). Finally, a control experiment with FTO_NU-1000, but no metal sulfide, yields a kinetic overpotential at 10 mA cm^−2^ of *ca*. 640 mV. Although the value in isolation is unremarkable, the *ca*. 360 mV decrease in *η* relative to the MOF-free bare FTO electrode is both substantial and unexpected. The isolated MOF contains no components capable of redox mediation and there are no obvious sites for adsorption and stabilization of potential reaction intermediates such as neutral hydrogen atoms.

Returning to the hybrid Ni-S catalyst, the observed kinetic overpotential at 10 mA cm^−2^ compares reasonably well with recently reported results for several other non-noble metal electrocatalysts for the HER in aqueous acid. For example, an NiS_2_ electrocatalyst showed only 4 mA cm^−2^ at around 240 mV of overpotential, Ni_3_S_2_ exhibit 10 mA cm^−2^ at 213 mV of overpotential, whereas CoS_2_ and FeS_2_ yielded 10 mA cm^−2^ at *η*-values of *ca*. 230 and 260 mV, respectively (all in 0.5 M H_2_SO_4_, pH 0; it is noteworthy that pH 1 was used in this work)^13^,^24^. In neutral (pH 7) solutions, *η*-values of <160 mV, at 10 mA cm^−2^, have been reported for electrodeposited and subsequently annealed CoS^46^.

The Ni-S mass loading on NU-1000_Ni-S films during electrodeposition was measured *in situ* using electrochemical quartz crystal microbalance (EQCM) techniques (see Methods section). As can be seen in Supplementary Fig. 6a, after 2 min of electrodeposition, Ni-S loading in NU-1000_Ni-S is 28 μg cm^−2^ (0.116 μmol cm^−2^). Potentiostatic electrolysis of NU-1000_Ni-S for a period of over 2 h at overpotential of 210 mV was done, while recording the charge flowing through the system (Supplementary Fig. 6b). From this charge, we have been able to estimate the amount of H_2_ formed during the electrolysis (taking into account the H_2_ Faradic yield of 93%, as determined by gas chromatography based on electrolysis at 2 mA cm^−2^ for 3 h; see Supplementary Fig. 7). We note that following previous reports, we suggest that Faradaic efficiency falls short of 100% due to H_2_ bubble formation on the surface of the electrode^15^,^47^. If we assume that only the portion of the metal sulfide that is in contact with the solution is catalytic (that is, the electrolyte does not permeate and swell the metal sulfide), and if we assume that an adjacent pair of nickel-coordinated sulfide ion constitutes one HER catalytic site, we obtain an active site normalized (upper limit) turnover frequency (TOF) of 34 s^−1^ and a turnover number (TON) of 273,000 (based on 8,000 s of electrolysis at a constant potential of −210 mV versus RHE; see Methods section for details). For the total amount of Ni-S in the NU-1000_Ni-S film (0.116 μmol cm^−2^), TOF and TON values were calculated to be 0.208 s^−1^ and 1668, respectively.

**Figure 5:**
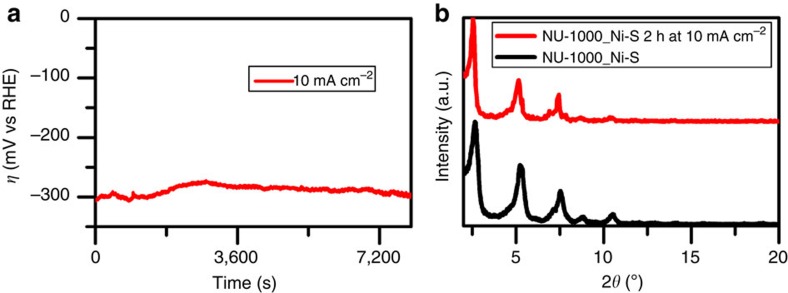
Stability analysis of NU-1000_Ni-S under HER working conditions. (**a**) Galvanostatic electrolysis measurement of NU-1000_Ni-S system under a constant current of 10 mA cm^−2^, showing the voltage stability of the system for more than 2 h. (**b**) Powder XRD plots for an NU-1000_Ni-S film before and after galvanostatic electrolysis measurement under a constant current of 10 mA cm^−2^ for 2 h. The plots show that framework crystallinity is retained.

As a preliminary test of the stability of the NU-1000_Ni-S combination, galvanostatic electrolysis measurements were conducted at 10 mA cm^−2^, in aqueous HCl at pH 1 (Fig. 5a). Notably, the overpotential needed to produce the imposed current remains constant (±20 mV) for 2 h of measurement, indicating that the NU-1000_Ni-S system can sustain its catalytic activity for relatively long periods of time. Power XRD measurements taken on a hybrid-catalyst film before and after 2 h of galvanostatic electrolysis at 10 mA cm^−2^ show that the MOF scaffold retains its crystallinity under electrocatalysis working conditions (Fig. 5b). To further assess the stability of the hybrid film during catalysis, we recorded a ultraviolet–visible spectrum of the pH 1 electrolyte solution after 2 h of galvanostatic electrolysis at 10 mA cm^−2^ (Supplementary Fig. 8), monitoring the wavelengths at which 1,3,6,8-tetrakis(p-benzoic acid)pyrene (H_4_TBAPy) linkers exhibit maximum absorbance (∼390 nm). The electrolysis solution displays no traces of leached NU-1000 linker, indicating that the MOF remains intact over the course of 2 h of catalysis. (For comparison, a spectrum of a digested NU-1000_Ni-S film is presented, showing a peak at 390 nm attributable to the linker.)

### Origin(s) of enhanced catalytic performance by hybrid assemblies

It is well known that in certain cases Tafel slopes can serve as indicators of the rate-determining step in the HER, as well as indicate the existence of other kinetically relevant effects^48^. Figure 4b shows Tafel plots (*V* versus log *J* plots) for the four types of electrodes examined here. For the MOF-free electrodes, with or without Ni-S, the slopes of the plots are near 180 mV per decade of current density, whereas for the NU-1000-functionalized electrodes the slopes are near 120 mV dec^−1^. Values substantially >120 mV typically are indicative of the presence of an uncompensated resistive element—for example, the resistance of the catalyst itself^49^.

If we assume that in the presence of the MOF scaffold, complicating factors are absent and the Tafel slope reflects only interfacial kinetics, the observed values of *ca*. 120 mV dec^−1^ imply that the HER is governed by either the Volmer–Heyrovsky or Volmer–Tafel mechanism (see Supplementary Note 1 for HER mechanistic details). Both mechanisms involve rate-limiting reduction of a proton to yield a catalyst-adsorbed H atom. For the Volmer–Heyrovsky mechanism, this step is followed by fast electrochemical reduction of a second proton and concomitant formation of an H–H bond. For the Volmer–Tafel pathway, the initial step is followed by fast formation of an H–H bond between a pair of surface-bound hydrogen atoms to form H_2_. It is noteworthy that the latter mechanism requires the catalyst to offer an immediately proximal pair of H-atom adsorption sites—for example, a pair of surface-exposed sulfide ions.

**Figure 6:**
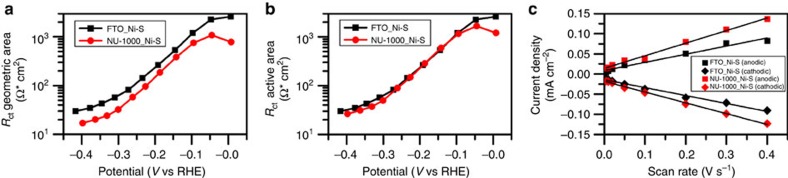
EIS analysis of the MOF's scaffold impact on the electronic properties of Ni-S. (**a**) A plot of geometric area normalized charge transfer resistance (*R*_ct_) versus applied potential, comparing between FTO_NiS and NU-1000_Ni-S electrodes. (**b**) A plot of electroactive surface area normalized *R*_ct_ versus applied potential, exhibiting similar *R*_ct_ values for both FTO_Ni-S and NU-1000_Ni-S electrodes. (**c**) A plot of double-layer capacitive current versus CV scan rate. The slope of each curve is relative to the amount of electroactive surface area of the system.

Still to be answered is why MOF-Ni-S hybrid catalyst formation decreases the overpotential for Ni-S-catalysed H_2_ evolution at 10 mA cm^−2^ by more than 200 mV. Our initial hypothesis, dispelled by SEM and EDS results mentioned above, was that catalyst electrodeposition within the MOF scaffold would greatly boost its surface area. Nevertheless, the presence of the MOF might still serve to roughen the electrodeposited film and therby increase its effective surface area. Cyclic voltammetry (CV) measurements of electrochemical currents in the voltage range positive of the hydrogen evolution region, that is, capacitive currents associated with electrical double-layer charging, are expected to scale as the solution-accessible surface area of the electrocatalyst^50^. Comparison of these currents over a range of voltammetric sweep rates (Fig. 6c) indicates remarkably similar surface areas. Indeed, the electroactive surface area for the hybrid assembly is only about 1.5 times greater than that for the simple Ni-S film, that is, far too little to account for the change in overpotential.

If the number of electrocatalytically active sites is little changed by introduction of the MOF scaffold, we are left with alterations in the local reaction environment as the most probable source of the observed reactivity enhancement. The putative local environment effects could conceivably take the form of electronic modulation of the properties of Ni-S, perhaps involving the creation of highly catalytic sites at MOF-engendered grain boundaries. Such effects could be especially important for two-dimensional layered compounds such as MoS_2_, but seem less likely to enormously influence the activity of an amorphous, three-dimensional material such as Ni-S.

To gain insight into the possible importance of electronic effects on the hybrid system HER performance, we employed electrochemical impedance spectroscopy with FTO_Ni-S and NU-1000_Ni-S electrodes, taken under HER working conditions. Nyquist plots show a resistance element which is attributed to solution voltage drops, in series with one semicircle corresponding to the parallel contribution of both catalyst's chemical capacitance and charge transfer resistance (*R*_ct_) at the catalyst/solution interface (see Supplementary Fig. 9)^15^. Figure 6 presents plots of log *R*_ct_ versus *V* for the two types of electrodes without (Fig. 6a) and with (Fig. 6b) correction for the minor difference in catalyst surface area. The plots show in striking manner that the interfacial electron-transfer step, in isolation, is not influenced by the presence of the MOF scaffold.

**Figure 7:**
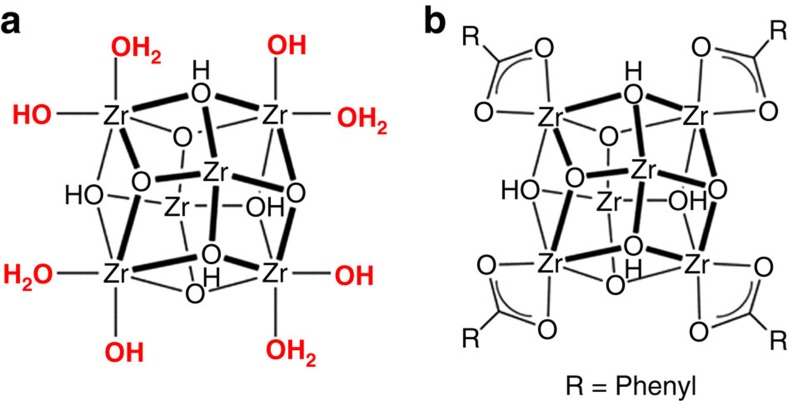
Effect of Zr_6_ based node's terminal –OH and –OH_2_ ligands on HER activity. (**a**) NU-1000's Zr_6_ based node, containing the terminal –OH and –OH_2_ ligands (marked in red). (**b**) Schematic representation of a benzoate-modified NU-1000 Zr_6_ based node. The benzoate replaces the node's terminal –OH and –OH_2_ ligands. Omitted, for simplicity, are atoms associated with coordinated linkers.

We next considered the possibility that catalytically important modification of the active-site environment could be assosiated with the solution side of the interface. The aquo- and hydroxo-rich nodes of the MOF (see Fig. 7a) could, for example, alter the local proton activity, as distinct from the bulk solution activity, or the MOF's nodes might facilitate local proton delivery and/or long-range proton transport.

**Figure 8:**
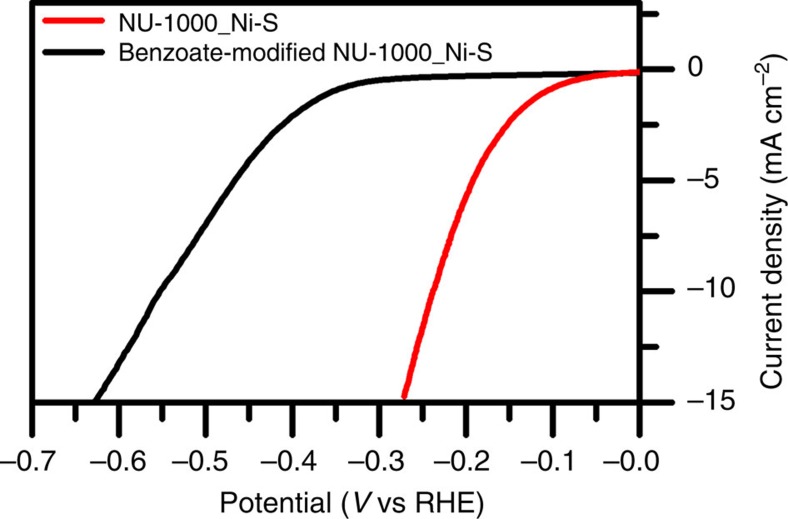
Effect of Zr_6_ based node's terminal –OH and –OH_2_ ligands, *J*–*V* curves. Comparison between the catalytic performance of NU-1000_Ni-S (red) and benzoate-modified NU-1000_Ni-S (black). Substitution of the terminal –OH and –OH_2_ ligands by benzoate ligands significantly reduces the HER catalytic activity of the hybrid film.

Although we lack specific insight into about how the aquo- and hydroxo-functionalized MOF node might assist Ni-S in catalysing the HER, we reasoned that largely eliminating these ligands could provide an indication of their importance. We assembled a variant of NU-1000_Ni-S using ‘as-synthesized' NU-1000 in which benzoate ligands replace the node's terminal –OH and –OH_2_ ligands^25^,^41^,^51^,^52^ (see Fig. 7b for Zr_6_-based node structure illustration and Supplementary Fig. 10 for ^1^H-NMR characterization of benzoate-modified NU-1000, showing the incorporation of four benzoates per Zr_6_ node). As shown by the *J*–*V* comparisons in Fig. 8, benzoate substitution eliminates the co-catalytic behaviour of the MOF scaffold, now requiring an overpotential of 553 mV at 10 mA cm^−2^. SEM images (top view and cross-section) of the benzoate-modified NU-1000 show that the MOF's crystal morphology has not changed and the film exhibits similar inter-rod spacing for the electrodeposition of Ni-S (Supplementary Fig. 11a). In addition, EQCM data shown on Supplementary Fig. 11b reveal similar mass loadings of Ni-S in the benzoate-modified NU-1000 film (26.7 μg cm^−2^) compared with aquo- and hydroxo-functionalized NU-1000 (28 μg cm^−2^). The results in Fig. 8 clearly demonstrate the importance of the node's terminal –OH and –OH_2_ ligands to the HER performance of the hybrid system. As one would anticipate, re-installing benzoate ligands on the MOF nodes and thereby displacing node-coordinated aqua and hydroxo ligands^51^,^52^,^53^ reverse the catalytic enhancement and yield HER *J*–*V* behaviour similar to that of the benzoate-containing ‘as-synthesized' version of the hybrid metal-sulfide/MOF system; see Supplementary Fig. 12.

**Figure 9:**
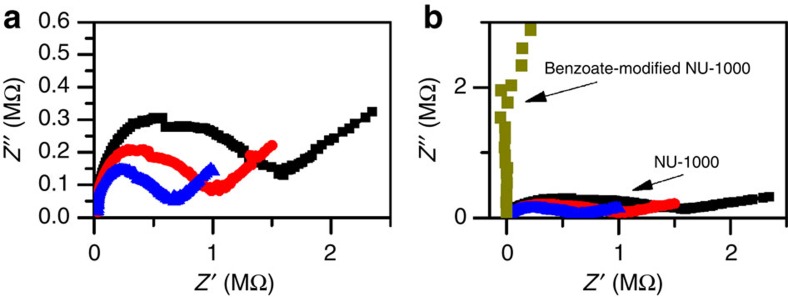
Proton conductivity measuremets. Nyquist plots of AC impedance measurements of proton conductivity of (**a**) NU-1000 in contact with humid air. As exposure time increases, the arc size decreases. (**b**) Comparison between Nyquist plots of NU-1000 and benzoate-modified NU-1000. The observed large resistance for the benzoate-modified sample is equivalent to low conductivity.

To gauge whether the aquo-ligated version of the MOF scaffold has the potential to assist proton transport, we examined its proton conductivity via impedance spectroscopy during and after infiltration with water from a high humidity atmosphere. A substantial literature on proton conductivity in MOFs now exists^54^,^55^,^56^,^57^. One means of engendering conductivity in MOFs is by the presentation of Bronsted acids or bases to an infiltrating hydroxylic solvent. Although p*K*_a_ and p*K*_b_ values have yet to be determined for the poly-protic nodes of NU-1000, clearly there is the potential for acid/base reactivity. Figure 9a shows representative Nyquist plots for a measurement of a pellet of NU-1000. The plots are characterized by a single arc, attributable to proton conductivity. The diameter of the arc decreases with time, reflecting progressive uptake of water and indicating increasing conductivity. The conductivity obtained at equilibrium is 2 × 10^−7^ S cm^−1^. Although of only modest magnitude, the observed conductivity implies the ability of the scaffold to assist in proton delivery to proximal catalytic sites. The extent to which such assistance is of value in aqueous 0.1 M HCl remains to be determined. In comparison with NU-1000, Nyquist plots for a measurement of a pellet of benzoate-modified NU-1000 (Fig. 9b) revealed such a significant increase in the arc's diameter (larger resistance or smaller conductivity) that no meaninful data fitting could be obtained. These measurements confirm that the node's terminal –OH and/or –OH_2_ ligands are essential for obtaining measurable proton conductivity.

## Discussion

In conclusion, hybrid electrocatalysts consisting of electrodeposited Ni-S and solvothermally grown, electrode-supported NU-1000, a mesoporous, acid-stable MOF, display very good activity for hydrogen evolution. In aqueous HCl at pH 1, the hybrid material can deliver a catalytic current of 10 mA cm^−2^ at an iR-corrected overpotential of just 238 mV—a sizable (*ca*. 200 mV) decrease in overpotential relative to MOF-free Ni-S films as electrocatalysts. The hybrid probably catalyses the reaction via the Volmer–Tafel mechanism, as evidenced in part by Tafel slopes near 120 mV dec^−1^ for plots of log *J* versus overpotential. Despite its substantial porosity and high surface area, the MOF scaffold does not exert its co-catalytic effect by boosting the electroactive surface area of the subsequently deposited nickel sulfide. Instead, it serves to modify the immediate environment of the electrocatalyst, rendering it more favourable for local proton delivery and/or transport. Thus, the aquo- and hydroxo-rich nodes of the MOF appear to contribute in a manner that is reminiscent of organic acids or bases in proximity to metal-ion-based cofactors in enzymes that catalyse proton-coupled electron-transfer reactions, a concept that has likewise been exploited in the design of abiotic molecular catalysts for both hydrogen^58^,^59^,^60^,^61^ and oxygen^62^ evolution, as well as CO_2_ reduction^63^.

## Methods

### Chemicals

All chemicals, benzoic acid (Aldrich, 99.5%), zirconyl chloride octahydrate (Aldrich, 98%), *N*,*N*-dimethylformamide (DMF) (Macron, 99.8%), hydrochloric acid (HCl, 36.5%–38.0%, Macron), acetone (Macron), NiCl_2_ (Aldrich, 98%) and thiourea (Sigma, 99%) were used as received without further purification. Deionized water was used throughout the work. The chemicals used for the synthesis of the H_4_TBAPy linkers were the same as those reported in our previous work^25^.

### Instrumentation

Thin-film XRD patterns were measured on a Rigaku ATX-G thin-film diffraction workstation.

SEM images and EDS mapping were measured on a Hitachi SU8030 SEM.

For ICP-OES experiments, two samples of the NU-1000_Ni-S thin film were scraped from their substrates and collected into a microwave vial (4 ml). Then, 0.25 ml concentrated H_2_O_2_ and 0.75 ml concentrated H_2_SO_4_ were added. The vial was capped and irradiated in a microwave oven at 150 °C for 5 min. The resultant clear solution was diluted to 25 ml with nanopure water and analysed via ICP-OES (Varian Vista MPX instrument). Ni, S and Zr concentration were calculated from external stock solutions.

Raman spectroscopy measurements were made using an Acton TriVista Confocal Raman Spectroscopy System. Sample irradiation was done with a 514.5-nm laser. The acquisition time was 60 s and the reported spectrum was obtained by averaging ten runs.

All CV and impedance spectroscopy experiments were performed on a Solarton Analytical Modulab Potentiostat equipped with an FRA Impedance module. A three-electrode electrochemical setup was used, with a platinum mesh counter electrode and Ag/AgCl/KCl (sat'd) electrode as reference electrode. Electrochemical data were measured in aqueous 0.1 M HCl (pH 1) solutions and were adjusted to RHE scale by adding (0.197+0.059 × pH) V to the measured potential. iR corrections were made to the obtained *i*–*V* curves according to the series resistances measured on the same electrochemical setup using impedance spectroscopy.

EQCM experiments were conducted with a MAXTEK RQCM system. The mass loading of Ni-S during electrodeposition was measured on a NU-1000-covered (by the recently described Electrophoretic deposition), tin-oxide-coated gold QCM crystal (6 MHz).

The Faradiac efficiency for H_2_ was determined by galvanostatic electrolysis at 2 mA cm^−2^ for 3 h. The experiment was carried out in a 60-ml Gamry 5-neck cell equipped with 3 Ace-Thred ports to hold each electrode and two joints capable of being sealed with septa for gas sparging. This setup included the NU-1000_Ni-S as the working electrode (*ca*. 0.5 cm^2^ surface area), a Pt wire counter electrode (flame annealed with a butane torch before use and separated from the bulk solution by fine glass frit) and a Ag/AgCl reference electrode (leakless assembly, eDAQ). Outside of the electrolyte solution, a bare portion of the FTO working electrode was attached to a Cu wire by a minimal amount of non-conductive, chemically resistant epoxy, to attach the FTO glass to the potentiostat leads. A BASi Epsilon potentiostat was used to apply constant current and record potential. The electrolysis experiment was carried out in 30 ml of total electrolyte solution (0.1 M HCl in water). The solution was constantly stirred throughout the experiment. Gas analysis was performed using 1 ml sample injections on a Hewlett-Packard 7890A Series gas chromatograph with two molsieve columns (30 m × 0.53 mm ID × 25 μm film). Gas chromatography calibration curve were made by sampling known volumes of H_2_ gas.

Upper limit TON and TOF were estimated in the following manner: we assume that only the portion of the metal sulfide that is in contact with the solution is catalytic (that is, the electrolyte does not permeate and swell the metal sulfide). In addition, we assume that an adjacent pair of nickel-coordinated sulfide ions in the Ni_3_S_2_ catalyst constitutes one HER catalytic site. Using the reported sulfur to sulfur distance in Ni_3_S_2_ (3.5 Å) as well as the ionic radius of S (1.7 Å), a rough estimate of catalytic site area in cm^2^ was made (assuming a rectangular shaped site):

Catalytic site length (3.5 × 10^−8^+(1.7 × 10^−8^ × 2)) × catalytic site width (1.7 × 10^−8^ × 2)=catalytic site area (2.34 × 10^−15^ cm^2^). The number of catalytic sites per 1 cm^2^ is 4.26 × 10^+14^; hence, there are 7.07 × 10^−10^ moles of catalytic sites per 1 cm^2^. The number of moles of produced H_2_ during 8,000 s of electrolysis (taking into account 93% H_2_ Faradaic efficiency) was calculated to be 1.94 × 10^−4^.

As a result, upper limit TON=moles of H_2_/moles of catalytic sites (273,000).

Upper limit TOF=upper limit TON/time of electrolysis (34 s^−1^).

TON and TOF values based on the total amount of Ni-S in the film (28 μg cm^−2^, 1.16 × 10^−7^ moles) are 1,668 and 0.208, respectively.

Impedance spectroscopy measurements under HER working conditions were made using an AC voltage of 20 mV, with a frequency range of 500 kHz–50 mHz.

### Growth of NU-1000 thin films

The growth of NU-1000 films on glass-supported, transparent and electronically conductive, FTO electrodes (‘FTO_NU-1000') was done using a slightly modified version of a previously reported solvothermal route^26^. The FTO glass substrate (15 Ω^−2^, Hartford Glass), with a size of 2.5 × 1.25 cm, was washed in soapy water, ethanol and acetone by sonication for 15 min sequentially. Thereafter, the substrate was dried and soaked in a solution of 0.5 mM H_4_TBAPy in DMF at room temperature for 12 h. The detailed synthesis of the H_4_TBAPy has been reported in our previous work^1^. The substrate was then cleaned with DMF and dried. Benzoic acid (2.7 g) and 105 mg of zirconyl chloride octahydrate were added into 8 ml of DMF and ultrasonically dissolved in a 20-ml screw-thread sample vial (Cole-Parmer, 28 mm × 57 mm), equipped with a urea cap and polytetrafluoroethylene (PTFE) foam-backed liner. Thereafter, the solution was placed into an oven at 80 °C for 2 h. After cooling down the solution to room temperature, 40 mg of H_4_TBAPy was added into this solution and the mixture was sonicated for 20 min. The as-prepared FTO substrate was then placed into the solution, with the conducting side facing down to the bottom. Subsequently, the vial was placed on the bottom of a gravity convection oven (VWR symphony) with the temperature set at 90 °C; the bottom of the oven provided a temperature gradient inside the vial, which is required for the growth of the MOF thin film. The vial was taken out from the oven after 13 h of reaction and the FTO substrate was taken out from the vial. After removing all the precipitations on the back side of the substrate, the substrate was washed with DMF; a uniform pale yellow MOF thin film could be observed on the front side of the FTO substrate. Benzoates (modulator) coordinated in the obtained MOF thin film was then removed by the following activation process: 0.5 ml of 8 M hydrochloric acid aqueous solution was mixed with 13 ml of DMF; 0.05 ml of the obtained solution was then mixed with 49.95 ml of DMF to form the diluted acidic solution. The MOF thin film was then soaked in the diluted acidic solution in a 100 °C oven for 4 days. Then, the film was washed with acetone for several times and soaked in acetone for 1 day. After drying the film in air, the FTO_NU-1000 thin film was then obtained. In a few cases, the HCl treatment was omitted and the benzoate-coordinating version of the material was used instead.

### Electrodeposition of Ni-S

Potentiostatic electrodeposition of Ni-S on FTO_NU-1000 (‘NU-1000_Ni-S') and bare FTO (‘FTO_Ni-S') films was conducted according to a slightly modified version of a previously published procedure^23^. Briefly, an aqueous solution containing 10 mM NiCl_2_ and 0.5 M thiourea as nickel and sulfur sources, respectively, was used as the deposition bath. The electrodeposition was carried out in a standard three-electrode setup containing either FTO_NU-1000 or bare FTO as a working electrode, Ag/AgCl as a reference electrode and FTO as a counter electrode. To determine the potential at which the deposition should be done, CV measurements of the deposition solution with and without a sulfur source were recorded (see Supplementary Fig. 1). It is clear that on addition of the sulfur source, the reduction peak for Ni^2+^/Ni^0^ (Ni deposition) is shifted to more anodic potentials, from −1.35 to −1.1 V versus Ag/AgCl, where the shift is a result of the reaction between the deposited Ni and thiourea (sulfur source) to generate Ni-S^23^. As a consequence, all subsequent Ni-S electrodepositions were carried out at a fixed potential of −1.1 V versus Ag/AgCl.

### Proton conductivity measurements

To measure the MOF's proton conductivity, disk pellets of NU-1000 (only) or benzoate modified NU-1000 were prepared, with dimensions of 7 mm diameter and 3 mm thickness. Each side of the pellet was coated with conductive silver epoxy, which was used to anchor a pair of tin-coated copper wires as electrical contacts. Then, the pellet was placed in an oven set to 60 °C for half an hour, to cure the epoxy. The pellet was exposed to H_2_O vapour at ambient temperature and its impedance spectroscopy response based on an AC signal of 20 mV was recorded over the frequency range of 500 kHz to 0.5 Hz.

## Additional information

**How to cite this article:** Hod, I. *et al.* A porous, proton-relaying, metal-organic framework material that accelerates electrochemical hydrogen evolution. *Nat. Commun.* 6:8304 doi: 10.1038/ncomms9304 (2015).

## Supplementary Material

Supplementary Information Supplementary Figures 1-14, Supplementary Tables 1-2, Supplementary Note 1 and Supplementary References
