# Supplementary material for: A porous proton-relaying metal-organic framework material that accelerates electrochemical hydrogen evolution
Source: Nat Commun. 2015 Sep 14;6:8304. doi: 10.1038/ncomms9304 (PMC4647847; doi:10.1038/ncomms9304)
Supplement: Supplementary Information — Supplementary Figures 1-14, Supplementary Tables 1-2, Supplementary Note 1 and Supplementary References [file ncomms9304-s1.pdf]

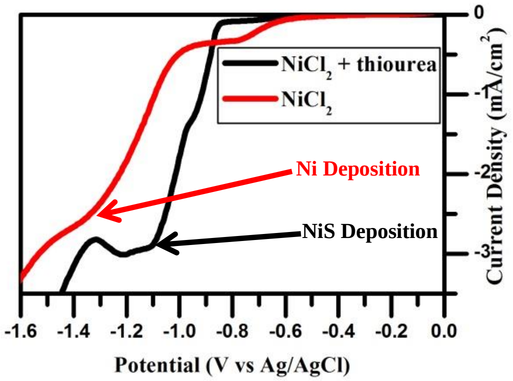

Supplementary Figure 1. Experimental conditions for the determination of Ni-S electrodeposition. The CV plot shows a comparison between a deposition bath containing only a Ni source and a deposition bath containing both Ni and S sources. The reaction between the reduced Ni and thiourea (S source) causes an anodic shift due to the deposition of Ni-S.

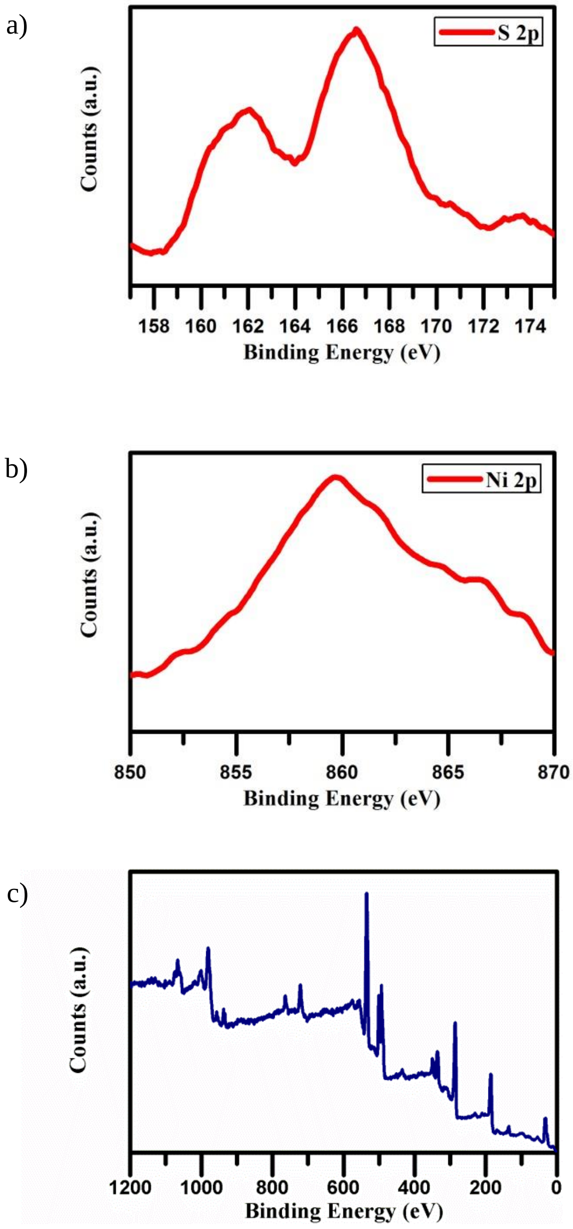

Supplementary Figure 2. X-ray photoelectron spectroscopy (XPS) measurement of the NU-1000_Ni-S film, showing fingerprint signal corresponding to S 2p (a) and Ni 2p (b) as well as the full XPS spectrum (c).

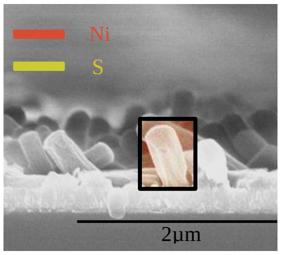

Supplementary Figure 3. EDS mapping of a single rod (rectangular area) was made, showing a trace amount of Ni and S along the NU-1000 rod.

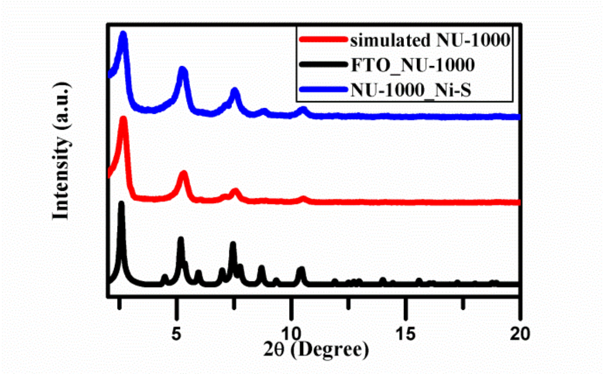

Supplementary Figure 4. PXRD patterns of simulated NU-1000, FTO_NU-1000 film and NU-1000_Ni-S film. Both films retain their NU-1000 crystal structure compared to the simulated one. In addition, due to its amorphous nature, there are no apparent peaks corresponding to Ni-S.

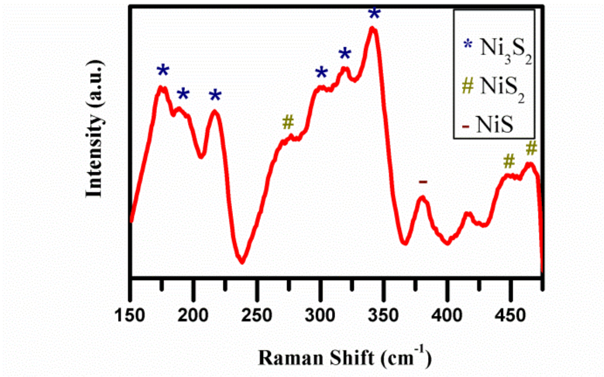

Supplementary Figure 5. Raman spectrum of an NU-1000_Ni-S sample, showing comparatively intense peaks for Ni_3S_2 main product, together with weaker peaks attributable to NiS_2 and NiS.

a)

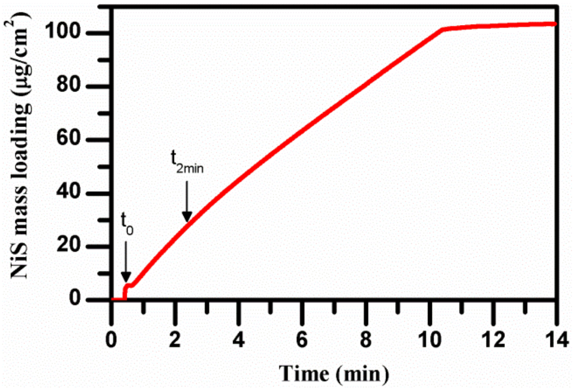

b)

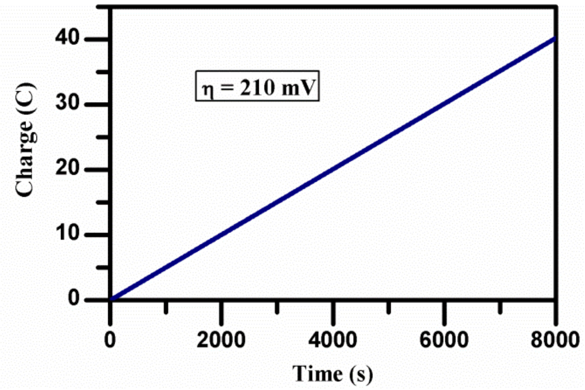

Supplementary Figure 6. a) Ni-S mass loading on NU-1000_Ni-S film as a function of electrodeposition time, measured in-situ using Electrochemical Quartz Crystal Micro-balance (EQCM) setup. b) Potentiostatic electrolysis of NU-1000_Ni-S film in pH 1 solution at Ƞ = 210 mV, showing the accumulated charge that passed through HER.

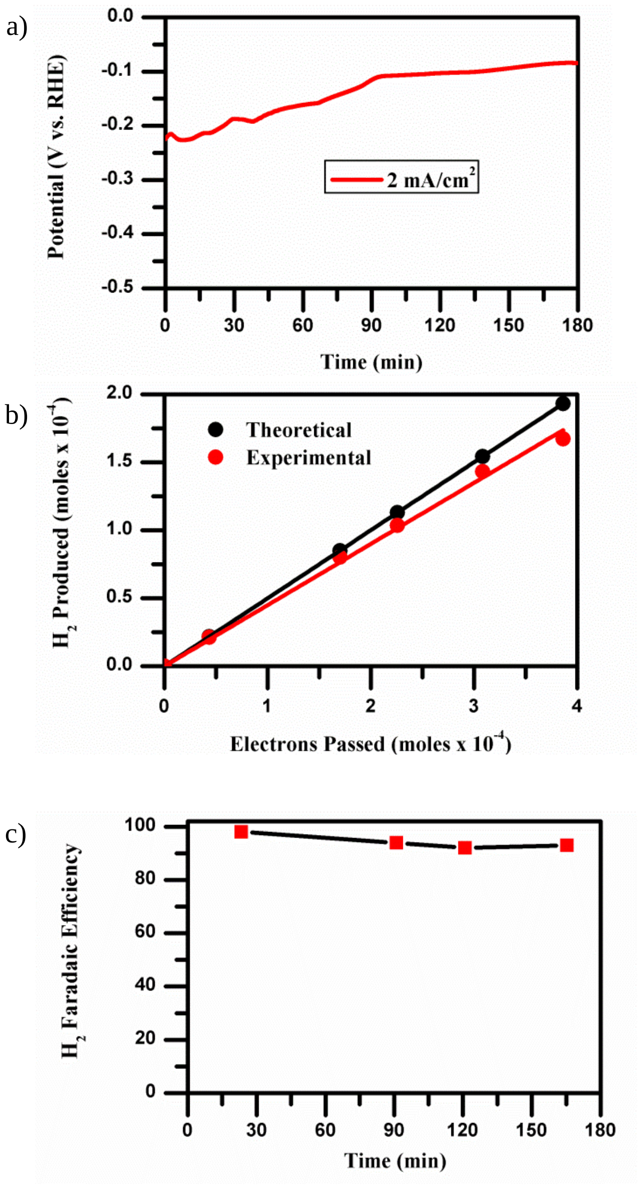

Supplementary Figure 7. a) Potential vs time plot during bulk electrolysis experiment at 2 mA/cm^2 for NU-1000_Ni-S; b) comparison between theoretical and experimental H_2 production during electrolysis; c) H_2 Faradaic efficiency for NU-1000_Ni-S.

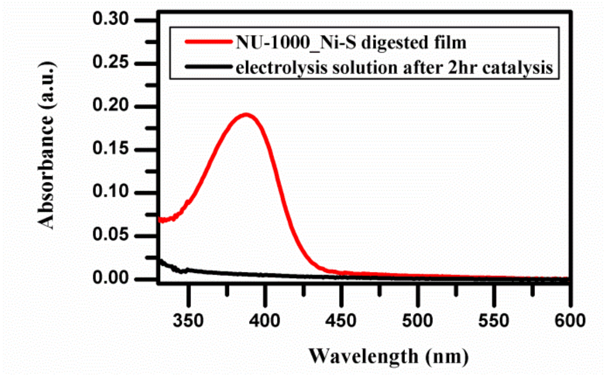

Supplementary Figure 8. UV-Vis spectroscopy of the galvanostatic electrolysis solution after 2 hours of operation at 10 mA/cm^2. The spectrum shows no trace for leached H_4TBAPy NU-1000 linker from the hybrid film. for comparison, a digested NU-1000_Ni-S film exhibits a peak centered at 390 nm, corresponding to the absorbance of the H_4TBAPy linker.

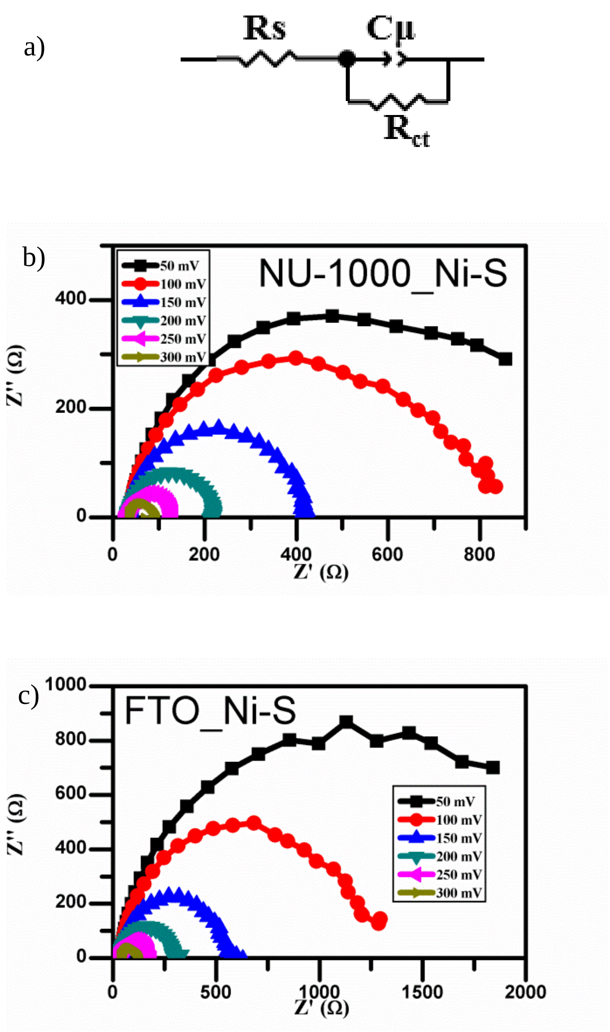

Supplementary Figure 9. Electrochemical impedance of FTO_Ni-S and NU-1000_Ni-S systems under HER conditions a) equivalent circuit used for fitting the data b) Nyquist plots of NU-1000_Ni-S system c) Nyquist plots of FTO_Ni-S system.

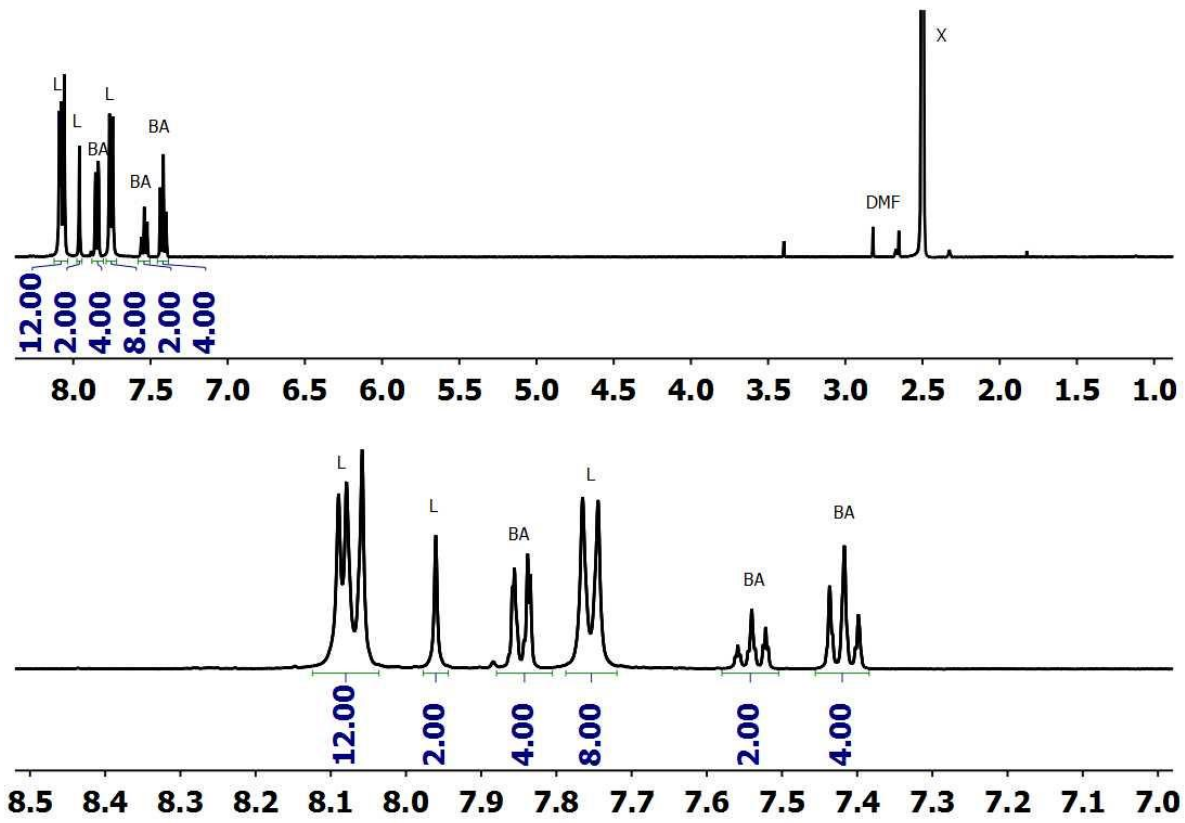

Supplementary Figure 10. ^{1}H NMR of benzoate modified NU-1000 sample. Integration of the peaks corresponding to benzoic acid (BA) and H_4TBAPy linker (L) shows a ratio of 2 benzoic acids per H_4TBAPy. Knowing that there are 2 H_4TBAPy linkers per Zr6 node, we can estimate 4 benzoic acids per Zr6 node.

a)

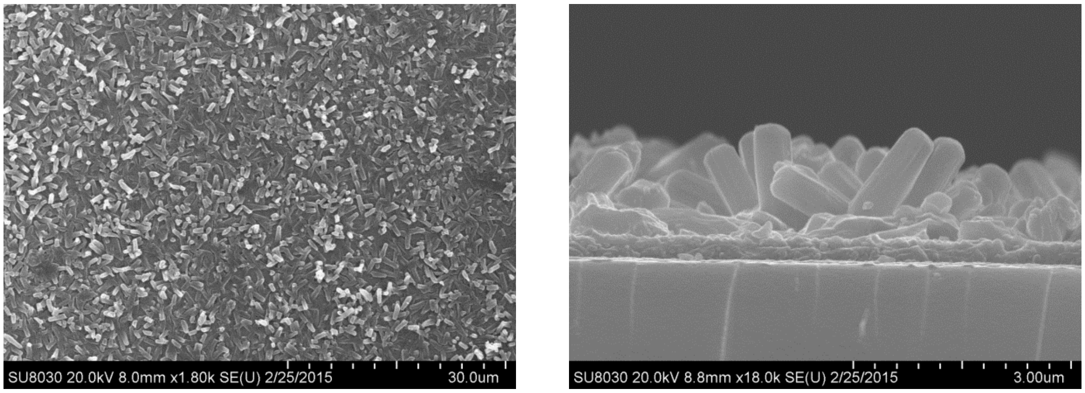

b)

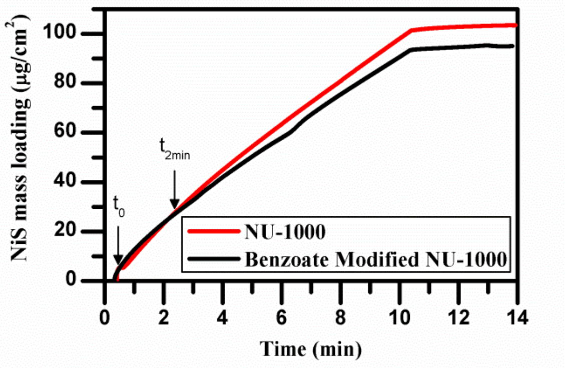

Supplementary Figure 11. a) Top view and cross-sectional SEM images of benzoate modified NU-1000. The images show the both the MOF morphology and inter-pillar spacing remains similar to the NU-1000 films b) EQCM comparison between Ni-S electrodepositiom on NU-1000 and on Benzoate modified NU-1000. In both cases, Ni-S mass loading is similar after 2 min electrodeposition (26.7 μg/cm^2 for Benzoate modified NU-1000 compared to 28 μg/cm^2 for NU-1000).

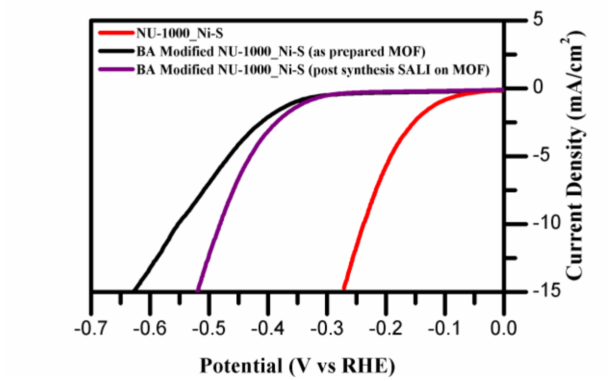

Supplementary Figure 12. J-V measurements. Comparison between benzoate-free (NU-100_Ni-S), as-prepared benzoate-modified NU-1000_Ni-S and SALI-modified[1-2] NU-1000_Ni-S.

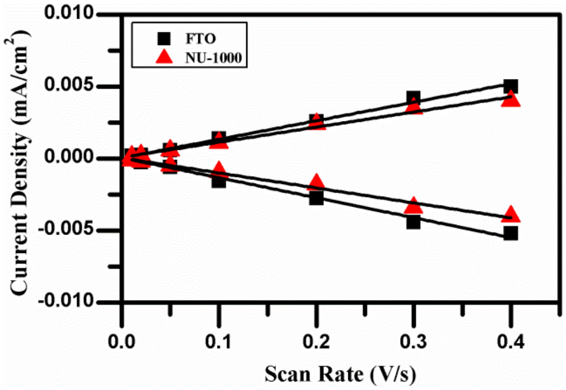

Supplementary Figure 13. Comparison between the Relative Electro-active surface area of bare FTO and NU-1000 electrodes, measured using double-layer electrochemical capacitance. The results show practically similar active surface area for both samples.

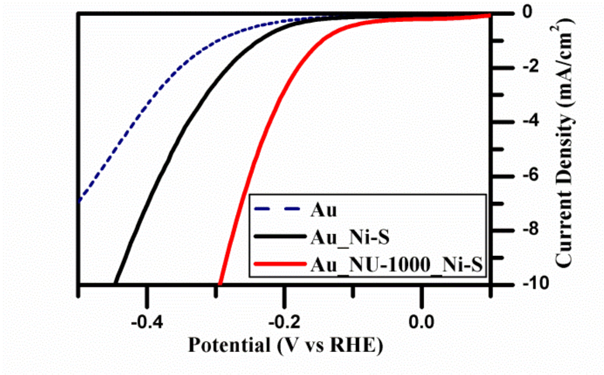

Supplementary Figure 14. J-V Comparison between bare Au, Au_Ni-S and Au_NU-1000_Ni-S. Even when FTO substrate is replaced by Au, similar HER performance is observed for the MOF hybrid system.

	EDS	ICP
Zr_6 : Ni	2.0 : 1.0	1.6 : 1.0
Ni : S	1.7 : 1.0	1.6 : 1.0

Supplementary Table 1. A table summarizing the elemental characterization of the NU-1000_Ni-S system (2 min electrodeposition), using EDS and ICP methods.

Sample	Ni content per film (ppm/cm^2)
**Benzoate-modified FTO_NU-1000**	**13.8**
**Benzoate-free FTO_NU-1000**	**13.6**

Supplementary Table 2. ICP measured Ni content (ppm) per MOF film, Comparison between benzoate-modified and benzoate-free FTO_NU-1000 films. PPM (parts-per-million) refers to the concentrations of dissolved nickel in solutions of identical volume used for ICP measurements.

Supplementary Note 1. Hydrogen Evolution Reaction Mechanisms:[3]

Three possible rate limiting steps have been suggested for the electrochemical hydrogen evolution reaction (HER) in acidic conditions.

I. Volmer step: $H^+ + e^- \rightarrow H_{ads}$

$$Tafel\ slope\ (b) = \frac{2.3RT}{\alpha F} \approx 120\ mV \quad .1$$

II. Heyrovsky step: $H_{ads} + H^+ + e^- \rightarrow H_2$

$$Tafel\ slope\ (b) = \frac{2.3RT}{(1+\alpha)F} \approx 40\ mV \quad .2$$

III. Tafel step: $2H_{ads} \rightarrow H_2$

$$Tafel\ slope\ (b) = \frac{2.3RT}{2F} \approx 30\ m\text{V} \quad .3$$

Where R is the ideal gas constant, T is the absolute temperature, F is the Faraday Constant and $\alpha \approx 0.5$ is the symmetry coefficient. The Tafel slope is determined according to the rate limiting step, which could be any one of the 3 steps mentioned above (30, 40 or 120 mV/dec).

For example, in the case of Pt electrocatalyst, it is known that the coverage of adsorbed hydrogens H_{ads} is very high. As a result, the HER is limited by the recombination of to surface bound hydrogens (Tafel step) and the Tafel slope is 30 mV/dec.

In this work, the obtained Tafel slope for the NU-1000_Ni-S system is close to 120 mV/dec. Hence, it implies for the fact that the rate of HER is limited by the Volmer step.

Supplementary References

1 Deria, P., Bury, W., Hupp, J. T. & Farha, O. K. *Chem. Commun.* **50**, 1965, (2014).

2 Deria, P., Mondloch, J. E., Tylianakis, E., Ghosh, P., Bury, W., Snurr, R. Q., Hupp, J. T. & Farha, O. K. *J. Am. Chem. Soc.* **135**, 16801, (2013).

3 Bockris, J. O. M. & Potter, E. C. The Mechanism of the Cathodic Hydrogen Evolution Reaction. *J Electrochem Soc* **99**, 169-186, (1952).
